# Pulmonary hypertension leads to poor inpatient outcome in non-white patients admitted with stroke: an analysis of national inpatient sample

**DOI:** 10.3389/fstro.2024.1350825

**Published:** 2024-02-08

**Authors:** Dilip Kumar Jayaraman, Stephanie Kjelstrom, Georgia Montone, Divya Rajasekaran

**Affiliations:** ^1^Department of Neurology, Main Line Health, Paoli, PA, United States; ^2^Main Line Health Center for Population Health Research, Wynnewood, PA, United States; ^3^SRM Medical College Hospital and Research Center, Kanchipuram, India

**Keywords:** stroke, morbidity, pulmonary hypertension, length of stay, atrial fibrillation

## Abstract

Stroke is one of the leading causes of death and disability worldwide. Every year, about 795,000 strokes are reported in the United States, of which around 23% are recurrent. We analyzed the national inpatient sample to assess the outcomes in patient with pulmonary hypertension and ischemic stroke. Our study included 7 million inpatient discharge encounters among which 553,085 patients had ischemic stroke. Among this, 16,830 had PH and 536,255 did not have PH.female (63.8% vs. 48.8%; *p* < 0.0001). A higher proportion of stroke patients with PH died in the hospital (5.7% vs. 3.7%; *p* < 0.0001) as well as had a longer average length of stay (LOS) [6.3 days (SD 6.2) vs. 5.0 days (SD 6.8); *p* < 0.0001]. Our study noted that black patients with PH were younger [70.5 years (SD 13.8)]. Black patients had the longest length of stay compared to Caucasians (7.8 days SD 8.3) (*p* < 0.0001).

## Introduction

Stroke refers to brain, spinal cord, or retinal cell death attributable to ischemia (Sacco et al., 2013). Stroke is one of the leading causes of death and disability worldwide. In 2020, about 7.08 million deaths worldwide were attributable to cerebrovascular disease. Every year, about 795,000 strokes are reported in the United States, of which around 23% are recurrent (Tsao et al., 2023).

Ischemic strokes can be sub-grouped based on the Trial of Org 10172 in Acute Stroke Treatment (TOAST) Criteria (Adams et al., 1993). Strokes are most commonly ischemic (Tsao et al., 2023) and, based on the TOAST criteria, can be subdivided into large artery atherosclerosis, lacunar strokes, cardioembolic, embolic strokes of undetermined source, and others. Risk factors can be both modifiable and non-modifiable. The most common modifiable risk factors are Hypertension, smoking, diet, and physical inactivity (Boehme et al., 2017).

As the pathophysiologic mechanisms of different subgroups of strokes are better studied, additional contributory causes are being postulated to cause strokes. One such uncommon association of stroke is pulmonary Hypertension (PH).

Pulmonary Hypertension encompasses a broad spectrum of disorders affecting the pulmonary vascular bed, resulting in increased pulmonary arterial pressure (Anderson and Lau, 2022). Progressive remodeling of the pulmonary vascular bed leads to right ventricle dysfunction. Pulmonary Hypertension is classified based on clinical features and pathophysiology, among which pulmonary Hypertension secondary to pulmonary disease and left heart diseases are much more common.

Pulmonary Hypertension is subdivided into five groups: Pulmonary arterial hypertension (PAH) (group 1), PH with left heard disease (group 2), PH- associated with lung diseases (group 3), chronic thromboembolic pulmonary hypertension (group 4), and unclear and multifactorial mechanisms (group 5) (Anderson and Lau, 2022). The prevalence varies among the subgroups; the prevalence in group 1 is 12.4–268 ppm (patients per million)while in group 4, the prevalence is 14.5–144 ppm (Leber et al., 2021). In one population study in the Netherlands, PH diagnosed by echocardiography has a prevalence of 2.6% (Moreira et al., 2015). The age-adjusted PH mortality rate was 7.9 per 100,000 individuals, and the study also noted an increase of 1.9% per year between 1999 and 2019 (Kang et al., 2022).

Careful assessment of medical history, physical examination, echocardiograms, and hemodynamic parameters (obtained via right-sided heart catheterization) is essential to diagnose and characterize the different forms of PH effectively.

### Aim

We reviewed the national inpatient sample database for risk Factors and Hospital Outcomes for Pulmonary Hypertension among patients with cerebral Infarction.

### Statistical methods

Over 7 million inpatient records are included in the 2019 National Inpatient Sample database. Patients with a cerebral infarction (CI) as their primary diagnosis (first discharge diagnosis) identified by ICD10 code I63^*^ were included in the analysis, an unweighted total of 110,617. Of the patients with a CI, we identified those with a pulmonary hypertension (PH) diagnosis on all but their first discharge diagnosis by ICD10 codes I27.0, I27.2, and I27.9. All patient and encounter characteristics were analyzed using standard descriptive statistics, frequency (percent) for categorical variables, and mean (standard deviation) for continuous variables. Variables were created to collapse race (White, Black, Hispanic, Asian or Pacific Islander, and Other) and to organize data into three categories based on weighted Elixhauser index scores (< 6, between 6 and 15, and over 15). We further identified comorbidities that are risk factors for CI from secondary discharge diagnoses such as tobacco use, congestive heart failure, diabetes, hypertension, atrial fibrillation, hyperlipidemia, obesity, and cancer. Comparisons were made between CI patients with PH and those without PH, and results were stratified by age, length of stay (LOS), hospital mortality, race, income level, type of insurance, sex, comorbidities, Elixhauser index scores, total charges, the population size of the hospital's city or town, and whether a patient had a major surgery during their admission. We used Pearson chi-square tests for categorical variables and two sample *t*-tests for continuous variables. We also isolated all CI patients with PH and compared them by race.

Multivariable logistic regression models were built to assess the risk factors of having PH. Additional univariable and multivariable hierarchical models were built to assess whether there was a difference between the PH and non-PH group for hospital mortality and LOS. For the latter models, model 1 was univariable, model 2 was adjusted for age, sex, and race, and model 3 was adjusted for variables in model 2 plus insurance, income, comorbidities, Elixhauser index, and rural/urban. We took the logarithm of LOS for the regression models to normalize the variable and for easier interpretation. The alpha level is 0.05, a *p* < 0.05 is considered significant, and 95% confidence intervals are reported. Analyses were performed using NIS strata and NIS discharge weights as probability weights. All analyses were conducted in Stata 17.0 (Statacorp, LLC., College Station, TX).

### Table 1 results

CI patients with PH were on average older[(76.7 years (SD 12.6) vs. 69.7 years (SD 14.0); *p* < 0.0001], female (63.8% vs. 48.8%; *p* < 0.0001), White (68.9% vs. 67.9%; *p* = 0.01), from areas with higher median income (20.1% vs. 18.9%, *p* = 0.023), and on Medicare (80.5% vs. 64.3%; *p* < 0.0001) compared to CI patients without PH. A higher proportion of CI patients with PH died in the hospital (5.7% vs. 3.7%; *p* < 0.0001) as well as had a longer average LOS [6.3 days (SD 6.2) vs. 5.0 days (SD 6.8); *p* < 0.0001], and they were also charged more [$82.4/$1,000 (SD $99.1) vs. $70.8/$1,000 (SD 95.7); *p* < 0.0001] compared to CI patients without PH. PH patients experienced a higher number of comorbidities than non-PH patients, with 55.8% of PH patients and 26% of non-PH patients having an Elixhauser score of 16 or above. Of the CI related comorbidities, compared to non-PH patients, a higher percentage of PH patients had hypertension with complications (61.7% vs. 28.7%; *p* < 0.0001), atrial fibrillation (55.9% vs. 24.6%; *p* < 0.0001), and CHF (56.2% vs. 17.9%; *p* < 0.0001). On the other hand, a higher percentage of non-PH patients had diabetes with no complications (16.2% vs. 11.7%; *p* < 0.0001), hypertension with no complications (56.5% vs. 29.4%; *p* < 0.0001), and used tobacco (20.5% vs. 11.4%; *p* < 0.0001). A higher percentage of PH patients compared to non-PH patients were more likely to have a major surgery (14.4% vs. 12.3%; *p* = 0.0007).

The data on obesity, cancer, metastatic cancer, and the locations of the patients' hospitals were not statistically significant (Table 1).

**Table 1:** All acute ischemic stroke patients.

**Weighted results**				
	**No PH**	**PH**	***p***	**Total**
	***n** =* **536,255**	***n** =* **16,830**		***n** =* **553,085**
Age (Mean/SD)	69.7 (14.0)	76.7 (12.6)	< 0.0001	69.9 (14.0)
Female *n* (%)	48.80%	63.80%	< 0.0001	49.20%
Race *n* (%)			0.01	
White	67.90%	68.90%		67.90%
Black	17.60%	18.90%		17.60%
Hispanic	8.20%	7.00%		8.20%
Asian or Pacific Islander	3.20%	2.80%		3.10%
Native American	0.50%	0.50%		0.50%
Other	2.70%	1.90%		2.60%
Hosp Mortality *n* (%)	3.70%	5.70%	< 0.0001	3.70%
Total Charges per $1000 (mean/SD)	$70.8 (95.7)	$82.4 (99.1)	< 0.0001	$71.2 (95.8)
LOS (days/mean/SD)	5.0 (6.8)	6.3 (6.2)	< 0.0001	5.0 (6.8)
Payer			< 0.0001	
Medicare	64.30%	80.50%		64.80%
Medicaid	9.50%	6.50%		9.40%
Private	19.30%	10%		19%
Self-Pay	4.30%	1.70%		4.20%
No charge	0.30%	0.00%		0.30%
Other	2.40%	1.30%		2.40%
Median household income			0.023	
$1–49,999	31.20%	28.50%		31.10%
$50k−64999	25.70%	26.70%		25.80%
$65k−85999	24.20%	24.70%		24.20%
$86k+	18.90%	20.10%		18.90%
**Comorbidities**				
Diabetes uncomplicated	16.20%	11.70%	< 0.0001	16.10%
Diabetes complicated	23.80%	26.00%	0.004	23.90%
Hypertension complicated	28.70%	61.70%	< 0.0001	29.70%
Hypertension uncomplicated	56.50%	29.40%	< 0.0001	55.70%
A Fib	24.60%	55.90%	< 0.0001	25.60%
Tobacco use	20.50%	11.40%	< 0.0001	20.30%
CHF	17.90%	56.20%	< 0.0001	19.10%
Hyperlipidemia	60.80%	60.50%	0.667	60.80%
Obesity	14.90%	15.90%	0.11	14.90%
Cancer	3.40%	3.40%	0.957	3.40%
Metastatic cancer	1.90%	1.60%	0.223	1.90%
Elixhauser index			< 0.0001	
< 6	30.20%	12.20%		29.70%
6–15	43.80%	31.90%		43.50%
> 15	26%	55.80%		26.90%
Urban/rural county metric			0.08	
Central Metro area > 1 mil	27.60%	27.40%		27.60%
Fringe Metro Area > 1 mil	24%	25.70%		24%
Metro area 250k−999,999	21.40%	21.50%		21.40%
Metro area 50k−249,999	9.90%	10%		9.90%
Micropolitan	9.80%	9.50%		9.80%
Not metro or micro	7.40%	5.90%		7.30%
Major surgery *n* (%)	12.30%	14.40%	0.0007	12.40%

### Table 2 results

White CI patients with PH were on average the oldest [78.6 years (SD 11.4)] while Black CI patients with PH were the youngest [70.5 years (SD 13.8)] out of the five racial groups (White, Black, Hispanic, Asian or Pacific Islander, and Other; *p* < 0.0001). The “Other” category includes Native Americans and other racial groups not otherwise listed. Most patients were female for all racial groups but did not differ between groups (*p* = 0.365). Although not statistically different, Hispanic patients were most likely to die at the hospital, while Asian or Pacific Islander patients were least likely (hospital mortality rates: 7% vs. 3.3%; *p* = 0.665). On average, White patients had the shortest LOS [5.7 days (SD 4.9) and Black patients had the longest (7.8 days SD 8.3) (*p* < 0.0001)]. Most patients, across all racial groups, had Medicare. However, a higher percentage of White patients (84.9%; *p* < 0.0001) were on Medicare than of any other group. In terms of income level, White patients were the most evenly spread out among the NIS's four median household income categories. Meanwhile, almost half (48.7%; *p* < 0.0001) of all Black patients fell into the lowest category and Asian or Pacific Islander patients were, on average, of the highest income levels. In terms of comorbidities, the greatest disparities existed between White (22%) and Hispanic (40.9%) patients who had diabetes with complications (*p* < 0.0001), White patients (10.6%) and patients in the “Other” racial category (17.7%) with uncomplicated diabetes (*p* = 0.021), Asian or Pacific Islander (46.2%) and Black (70.2%) and Hispanic (70%) patients who had hypertension with complications (*p* < 0.0001), Asian or Pacific Islander (42.9%) and Hispanic (22.6%) patients with hypertension without complications (*p* = 0.0001), Black (40.3%) and White (60.6%) patients with atrial fibrillation (*p* < 0.0001), Asian or Pacific Islander (3.3%) and Black (16.3%) patients who use tobacco (*p* = 0.0001), Asian or Pacific Islander (38.5%) and Black (64%) patients with CHF (*p* < 0.0001), and Asian or Pacific Islander (6.6%) and Black (20.3%) patients who are obese (*p* = 0.001). There was no significant difference between the groups for hyperlipidemia, cancer, or metastatic cancer. A lower percentage of Asian or Pacific Islander patients had an Elixhauser score > 15 compared to all other racial groups (*p* < 0.0001). A higher percentage of Black, Hispanic, and Asian or Pacific Islander patients were treated in big cities compared to White or Other races (*p* < 0.0001). The data on major surgeries was statistically insignificant (Table 2).

**Table 2:** All CI patients with ph by race and patient characteristics.

	**White**	**Black**	**Hispanic**	**Asian or Pacific Islander**	**Other**		**Total**
	***n** =* **11,310**	***n** =* **3,100**	***N*** = **1,150**	***n** =* **455**	***n** =* **395**	***p***	***n** =* **16,410**
Age (Mean/SD)	78.6 (11.4)	70.5 (13.8)	74.5 (14.4)	77.1 (11.7)	75.3 (12.8)	< 0.0001	76.7 (12.6)
**Sex** ***n*** **(%)**						0.365	
Male	4,035 (35.7%)	1,090 (35.2%)	480 (41.7%)	180 (39.6%)	145 (36.7%)		5,930 (36.1%)
Female	7,275 (64.3%)	2,010 (64.8%)	670 (58.3%)	275 (60.4%)	250 (63.3%)		10,480 (63.9%)
Mortality *n* (%)	660 (5.8%)	165 (5.3%)	80 (7%)	15 (3.3%)	15 (3.8%)	0.665	935 (5.7%)
LOS (Mean/SD)	5.7 (4.9)	7.8 (8.3)	6.9 (7.3)	7.2 (8.2)	7.2 (9.7)	< 0.0001	6.3 (6.2)
**Payer**						< 0.0001	
Medicare	9,600 (84.9%)	2,175 (70.2%)	815 (70.9%)	315 (69.2%)	325 (82.3%)		13,230 (80.6%)
Medicaid	380 (3.4%)	420 (13.6%)	205 (17.8%)	25 (5.5%)	35 (8.9%)		1,065 (6.5%)
Private	1,050 (9.3%)	355 (11.5%)	90 (7.8%)	90 (19.8%)	30 (7.6%)		1,615 (9.8%)
Self-Pay	135 (1.2%)	95 (3.1%)	30 (2.6%)	< 11	< 11		275 (1.7%)
No charge	< 11	< 11	< 11	< 11	< 11		< 11
Other	140 (1.2%)	50 (1.6%)	< 11	15 (3.3%)	< 11		215 (1.3%)
**Median household income**						< 0.0001	
$1–49,999	2,595 (23.2%)	1,495 (48.7%)	385 (34.5%)	60 (13.2%)	115 (30.7%)		4,650 (28.7%)
$50k−64999	3,140 (28.1%)	655 (21.3%)	295 (26.5%)	70 (15.4%)	95 (25.3%)		4,255 (26.3%)
$65k−85999	2,960 (26.5%)	565 (18.4%)	260 (23.3%)	160 (35.2%)	80 (21.3%)		4,025 (24.9%)
$86k+	2,490 (22.3%)	355 (11.6%)	175 (15.7%)	165 (36.3%)	85 (22.7%)		3,270 (20.2%)
**Comorbidities**							
Diabetes Uncomplicated	1,200 (10.6%)	450 (14.5%)	130 (11.3%)	70 (15.4%)	70 (17.7%)	0.021	1,920 (11.7%)
Diabetes complicated	2,490 (22%)	1,100 (35.5%)	470 (40.9%)	130 (28.6%)	115 (29.1%)	< 0.0001	4,305 (26.2%)
Hypertension complicated	6,725 (59.5%)	2,175 (70.2%)	805 (70%)	210 (46.2%)	240 (60.8%)	< 0.0001	10,155 (61.9%)
Hypertension uncomplicated	3,485 (30.8%)	730 (23.6%)	260 (22.6%)	195 (42.9%)	125 (31.7%)	0.0001	4,795 (29.2%)
A Fib	6,855 (60.6%)	1,250 (40.3%)	555 (48.3%)	265 (58.2%)	205 (51.9%)	< 0.0001	9,130 (55.64%)
Tobacco Use	1,230 (10.9%)	505 (16.3%)	90 (7.8%)	15 (3.3%)	45 (11.4%)	0.0001	1,885 (11.5%)
CHF	6,200 (54.8%)	1,985 (64%)	665 (57.8%)	175 (38.5%)	230 (58.2%)	< 0.0001	9,255 (56.4%)
Hyperlipidemia	6,840 (60.5%)	1,840 (59.4%)	710 (61.7%)	330 (72.5%)	215 (54.4%)	0.14	9,935 (60.5%)
Obesity	1,660 (14.7%)	630 (20.3%)	195 (17%)	30 (6.6%)	75 (19%)	0.001	2,590 (15.8%)
Cancer	395 (3.5%)	130 (4.2%)	25 (2.2%)	20 (4.4%)	< 11	0.286	570 (3.5%)
Metastatic Cancer	200 (1.8%)	50 (1.6%)	15 (1.3%)	< 11	< 11	0.769	270 (1.65%)
**Elixhauser index**						< 0.0001	
< 5	1,395 (12.3%)	365 (11.8%)	150 (13.0%)	55 (12.1%)	60 (15.2%)		2,025 (12.3%)
6–15	3,540 (31.3%)	990 (31.9%)	385 (33.5%)	195 (42.9%)	115 (29.1%)		5,225 (31.8%)
> 15	6,375 (56.4%)	1,745 (56.3%)	615 (53.5%)	205 (45.1%)	220 (55.7%)		9,160 (55.8%)
**Urban/Rural County Metric**						< 0.0001	
Central Metro area > 1 mil	2,300 (20.4%)	1,375 (44.5%)	525 (46.3%)	200 (44%)	135 (34.6%)		4,535 (27.7%)
Fringe Metro Area > 1 mil	3,090 (27.4%)	720 (23.3%)	215 (18.9%)	105 (23.1%)	85 (21.8%)		4,215 (25.8%)
Metro Area 250k−999,999	2,565 (22.7%)	520 (16.8%)	255 (22.5%)	100 (22%)	55 (14.1%)		3,495 (21.7%)
Metro Area 50k−249,999	1,310 (11.6%)	190 (6.2%)	80 (7.1%)	25 (5.5%)	30 (7.7%)		1,635 (10%)
Micropolitan	1,260 (11.2%)	180 (5.8%)	55 (4.9%)	15 (3.3%)	50 (12.8%)		1,560 (9.5%)
Not Metro or Micro	770 (6.8%)	105 (3.4%)	< 11	< 11	35 (9%)		925 (5.7%)
Major Surgery n (%)	1,515 (13.4%)	485 (15.7%)	200 (17.4%)	70 (15.4%)	65 (16.5%)	0.352	2,335 (14.2%)

### Table 3 results

Table 3 is a multivariable analysis to determine which CI patient characteristics are risk factors for PH. After adjustment, the following CI groups have a higher odds of a PH, older patients [OR 1.02 (1.02, 1.03)], females compared to males [OR 1.66 (1.5, 1.8)], Black patients compared to White patients [OR 1.30 (1.1, 1.5)], having Medicare or Medicaid compared to private insurance [OR 1.16 (1.01, 1.3), OR 1.29 (1.07, 1.6) respectively], and people with an Elixhauser index score of > 15 compared to those with scores of 5 or below [OR 1.28 (1.14, 1.4)]. In addition, patients diagnosed with atrial fibrillation have an almost 100% higher odds [OR 1.99 (1.8, 2.2)] and those diagnosed with congestive heart failure (CHF) have a 220% higher odds of being diagnosed with PH [OR 3.2 (2.9, 3.5)]. Patients diagnosed with hypertension with complications had 40% higher odds of being diagnosed with PH [OR 1.4 (1.3, 1.6)] and obese patients had almost 30% higher odds [OR 1.27 (1.1, 1.4)]. On the other hand, people with diabetes with complications have 10% lower odds of being diagnosed with PH [OR 0.9 (0.8, 0.99)]. Patients who received medical care in rural areas (i.e., not metropolitan or micropolitan areas) had 20% lower odds of being diagnosed with PH compared to those in central cities with more than 1 million residents [OR 0.8 (0.7, 0.99)]. All other data were not statistically significant.

**Table 3:** Multivariable logistic regression, risk factors for CI patients having a PH.

	**OR (95% CI)**	***p***
Age (Mean/SD)	1.02 (1.02, 1.03)	< 0.0001
Female	1.66 (1.5, 1.8)	< 0.0001
**Race**		
White	Ref	
Black	1.30 (1.1, 1.5)	< 0.0001
Hispanic	1.04 (0.9, 1.2)	0.641
Asian or Pacific Islander	1.05 (0.8, 1.3)	0.727
Native American	1.1 (0.6, 1.9)	0.74
Other	0.8 (0.6, 1.9)	0.173
**Payer**		
Medicare	1.16 (1.01, 1.3)	0.038
Medicaid	1.29 (1.07, 1.6)	0.008
Private	Ref	
Self-Pay	0.93 (0.7, 1.3)	0.617
No Charge	0.8 (0.6, 1.2)	0.299
**Other**		
**Median household income**		
$1–49,999	0.93 (0.8, 1.1)	0.311
$50k−64999	1.0 (0.9, 1.1)	0.956
$65k−85999	0.99 (0.9, 1.1)	0.859
$86k+	Ref	
**Comorbidities**		
Diabetes Complicated	0.9 (0.8, 0.99)	0.033
Hypertension complicated	1.4 (1.3, 1.6)	< 0.0001
A Fib	1.99 (1.8, 2.2)	< 0.0001
Tobacco use	0.92 (0.8, 1.0)	0.145
CHF	3.2 (2.9, 3.5)	< 0.0001
Obesity	1.27 (1.1, 1.4)	< 0.0001
**Elixhauser index**		
< 6	Ref	
6–15	1.07 (0.9, 1.2)	0.266
> 15	1.28 (1.14, 1.4)	< 0.0001
**Urban/rural county metric**		
Central metro area > 1 mil	Ref	
Fringe metro area > 1 mil	1.1 (0.9, 1.2)	0.262
Metro area 250k−999,999	1.1 (0.9, 1.2)	0.298
Metro area 50k−249,999	1.1 (0.9, 1.3)	0.235
Micropolitan	1.1 (0.9, 1.2)	0.396
Not metro or micro	0.8 (0.7, 0.99)	0.05
Major surgery *n* (%)	1.1 (0.97, 1.2)	0.134
LOS	1.01 (1.0, 1.0)	< 0.0001

### Table 4 results

To create Table 4, we ran three univariable and multivariable regression analyses to compare the probability of hospital mortality between CI patient with and without PH. In model 1, we found that patients with CI and PH had a 60% higher odds of dying while in the hospital compared to patients without PH [OR 1.6 (1.36, 1.88); *p* < 0.0001]. In the second model, after adjustment by age, sex, and race, the odds decreases to 35% [OR 1.35 (1.15, 1.59); *p* < 0.0001]. After full adjustment in model 3, the association between PH and hospital mortality is no longer significant.

**Table 4:** Univariable and multivariable models, probability of hospital mortality, comparison between CI patient with and without PH.

	**Model 1**		**Model 2**		**Model 3**	
	**OR (95% CI)**	***p***	**OR (95% CI)**	***p***	**OR (95% CI)**	***p***
No PH	Ref		Ref		Ref	
PH	1.6 (1.36, 1.88)	< 0.0001	1.35 (1.15, 1.59)	< 0.0001	0.97 (0.82, 1.15)	0.702
Age			1.03 (1.03, 1.03)	< 0.0001	1.02 (1.02, 1.03)	< 0.0001
Female			0.95 (0.89, 1.01)	0.105	0.98 (0.91, 1.05)	0.562
**Race**						
White			Ref		Ref	
Black			0.87 (0.78, 0.97)	0.01	0.87 (0.78, 0.97)	0.014
Hispanic			0.98 (0.85, 1.13)	0.756	0.98 (0.85, 1.14)	0.816
Asian or Pacific Islander			1.12 (0.93, 1.32)	0.253	1.11 (0.93, 1.33)	0.236
Native American			0.96 (0.61, 1.52)	0.867	0.82 (0.49, 1.39)	0.458
Other			1.22 (1, 1.48)	0.054	1.17 (0.95, 1.44)	0.144
**Payer**						
Medicare					0.8 (0.7, 0.91)	0.001
Medicaid					1.16 (0.99, 1.37)	0.069
Private					Ref	
Self-Pay					1.31 (1.07, 1.6)	0.007
No Charge					0.88 (0.38, 2.04)	0.762
Other					1.79 (1.36, 2.36)	< 0.0001
**Median household income**						
$1–49,999					1.06 (0.95, 1.19)	0.321
$50k−64999					0.98 (0.87, 1.1)	0.695
$65k−85999					1.02 (0.92, 1.14)	0.648
$86k+					Ref	
**Comorbidities**						
Diabetes complicated					0.93 (0.86, 1.01)	0.079
Hypertension complicated					0.68 (0.62, 0.75)	< 0.0001
A Fib					1.13 (1.05, 1.21)	0.001
Tobacco use					0.68 (0.61, 0.76)	< 0.0001
CHF					1.28 (1.16, 1.4)	< 0.0001
Obesity					0.84 (0.75, 0.95)	0.004
**Elixhauser Index**						
< 6					Ref	
6–15					2.0 (1.77, 2.26)	< 0.0001
> 15					5.86 (5.13, 6.69)	< 0.0001
**Urban/rural county metric**						
Central metro area > 1 mil					Ref	
Fringe metro area > 1 mil					1.04 (0.93, 1.17)	0.5
Metro area 250k−999,999					1.18 (1.05, 1.33)	0.005
Metro area 50k−249,999					1.16 (1, 1.34)	0.05
Micropolitan					1.28 (1.12, 1.47)	< 0.0001
Not metro or micro					1.4 (1.21, 1.63)	< 0.0001
Major surgery *n* (%)					2.12 (1.95, 2.3)	< 0.0001

### Table 5 results

To create Table 5, we ran three univariate and multivariate regression analyses to assess the relationship between LOS and PH for CI patients. We log-transformed the LOS variable, then raised *e* to the power of each beta coefficient, subtracted one, and multiplied by 100 to obtain percent changes [(*e*^β^ - 1) ^*^ 100]. In the unadjusted model 1, we found that patients with CI and PH on average had a 29.7% higher LOS compared to patients without PH [β = 0.26 (0.23, 0.29); *p* < 0.0001]. After adjustment by age, sex, and race in model 2, that percentage lowered slightly to 28.4%[(β = 0.25 (0.22, 0.28); *p* < 0.0001]. After full adjustment, the average patient with CI and PH had a hospital stay that was 10.5% longer than those without PH [β = 0.1 (0.07, 0.13); *p* < 0.0001].

**Table 5:** Univariable and multivariable linear regression to compare LOS for CI patients with and without PH.

	**Model 1**		**Model 2**		**Model 3**	
	β **(95% CI)**	***p***	β **(95% CI)**	***p***	β **(95% CI)**	***p***
No PH	Ref		Ref		Ref	
PH	0.26 (0.23, 0.29)	< 0.0001	0.25 (0.22, 0.28)	< 0.0001	0.1 (0.07, 0.13)	< 0.0001
Age			0.002 (0.002, 0.003)	< 0.0001	0.001 (0.001, 0.002)	< 0.0001
Female			0.006 (−0.003, 0.02)	0.188	0.02 (0.01, 0.03)	< 0.0001
**Race**						
White			Ref		Ref	
Black			0.23 (0.22, 0.25)	< 0.0001	0.18 (0.16, 0.2)	< 0.0001
Hispanic			0.11 (0.09, 0.14)	< 0.0001	0.06 (0.04, 0.08)	< 0.0001
Asian or Pacific islander			0.08 (0.04, 0.11)	< 0.0001	0.06 (0.24, 0.09)	0.001
Native american			0.02 (−0.05, 0.08)	0.608	−0.01 (−0.08, 0.05)	0.696
Other			0.14 (0.1, 0.18)	< 0.0001	0.1 (0.07, 0.14)	< 0.0001
**Payer**						
Medicare					0.04 (0.03, 0.06)	< 0.0001
Medicaid					0.24 (0.22, 0.26)	< 0.0001
Private					Ref	
Self-pay					0.08 (0.05, 0.11)	< 0.0001
No charge					0.15 (0.06, 0.24)	0.001
Other					0.05 (0.02, 0.09)	0.003
**Median household income**						
$1–49,999					0.07 (0.05, 1)	< 0.0001
$50k−64999					0.03 (0.01, 0.05)	0.006
$65k−85999					0.02 (−0.003, 0.04)	0.103
$86k+					Ref	
**Comorbidities**						
Diabetes complicated					0.1 (0.08, 0.11)	< 0.0001
Hypertension complicated					−0.02 (−0.03, −0.01)	< 0.0001
A Fib					0.003 (−0.01, 0.01)	0.656
Tobacco Use					−0.02 (−0.03, −0.01)	0.001
CHF					0.02 (0.004, 0.03)	0.013
Obesity					0.1 (0.09, 0.12)	< 0.0001
**Elixhauser index**						
< 6					Ref	
6–15					0.23 (0.22, 0.24)	< 0001
> 15					0.59 (0.57, 0.61)	< 0.0001
**Urban/rural county metric**						
Central metro area > 1 mil					Ref	
Fringe metro area > 1 mil					0.003 (−0.02, 0.02)	0.754
Metro area 250k−999,999					−0.02 (−0.04, 0.006)	0.156
Metro area 50k−249,999					−0.04 (−0.06, −0.01)	0.008
Micropolitan					−0.02 (−0.05, 0.003)	0.09
Not metro or micro					−0.02 (−0.05, 0.004)	0.096
Major surgery *n* (%)					0.55 (0.53, 0.56)	< 0.0001

## Discussion

Our study included 7 million inpatient discharge encounters among which 553,085 patients had ischemic stroke. Among this, 16,830 had PH and 536,255 did not have PH. We observed that patients with stroke and PH were, on average, older (76.7 years vs. 69.7 years) and, more commonly, female (63.8% vs. 48.8%; *p* < 0.0001). A higher proportion of stroke patients with PH died in the hospital (5.7% vs. 3.7%; *p* < 0.0001) as well as had a longer average LOS [6.3 days (SD 6.2) vs. 5.0 days (SD 6.8); *p* < 0.0001]. PH patients experienced a higher number of comorbidities than non-PH patients, with 55.8% of PH patients and 26% of non-PH patients having an Elixhauser score of 16 or above. Our study noted that black patients with PH were the youngest [70.5 years (SD 13.8)]. Black patients had the longest (7.8 days SD 8.3) (*p* < 0.0001). Moreover, after full adjustment, the average patient with stroke and PH had a hospital stay that was 10.5% longer than those without PH [β = 0.1 (0.07, 0.13); *p* < 0.0001]. In our analysis, patients diagnosed with atrial fibrillation have almost 100% higher odds [OR 1.99 (1.8, 2.2)], and those diagnosed with congestive heart failure (CHF) have 220% higher odds of being diagnosed with PH [OR 3.2 (2.9, 3.5)].

Pulmonary hypertension (PH) is a pulmonary vascular dysfunction with elevated mean pulmonary artery pressure < 20 mm Hg (Simonneau et al., 2019). Early diagnosis and initiation of treatment are crucial as it is estimated that an about 2-year delay in diagnosis is associated with increased disease burden (Maron, 2023). Signs and symptoms are often subtle and non specific. The most common symptoms are progressive dyspnea, fatigue and exhaustion, and decreased exercise capacity. Chest pain, syncope, and symptomatic heart failure can be seen in advanced situations. On exam, loud P2 component of second heart sound, jugular venous distension, S3 gallop, and murmur related to tricuspid regurgitation are present (Hoeper et al., 2017; Frost et al., 2019). The diagnosis is often delayed because of the nonspecific nature of the symptoms.

Initial clues to the diagnosis can be seen with a transthoracic echocardiogram showing increased tricuspid regurgitant jet velocity >2.8 m/s, suggesting elevated pulmonary artery systolic pressure (Maron, 2023). As with all the non-invasive measures, echocardiography has limitations, including suboptimal acoustic windows and the inability to estimate pulmonary artery wedge pressure.

*Stroke* is a complex neurovascular syndrome caused by varied causes. Effective management of ischemic stroke involves identifying risk factors and managing all the risk facts appropriately to prevent recurrence. About one in three strokes are recurrent and significantly impact patient and health care costs.

There is growing evidence of a relationship between pulmonary hypertension and ischemic stroke. A meta-analysis, including 32,523 patients, showed pooled prevention of stroke in patients with PH to be at 8% and 1.46 more significant risk of stroke in patients with PH than without PH (Shah et al., 2019). In another study using the NIS, PH was noted to independently increase the incidence of all-cause strokes in patients with Atrial fibrillation, leading to increased inpatient stay and mortality (Khattar et al., 2023). A previous study also noted higher inpatient mortality in male patients with pulmonary hypertension and ischemic stroke (Pana et al., 2021). In an observational study of patients with Atrial fibrillation, PH was more commonly associated with ischemic stroke (Karadavut and Cetin, 2022).

The exact pathophysiology is unclear, but possible mechanisms include underlying arteriopathy and hypercoagulable state. PH is a complex vasculopathy that can lead to multiple comorbidities, such as right heart failure and an increased stroke risk. Pulmonary hypertension is also associated with an increased risk of atrial fibrillation and risk of embolic events. Arteriopathy in PH resembles arteriopathy in ischemic stroke patients with sickle cell disease, suggesting shared physiology leading to proliferative vascular intimal and smooth muscle hypertrophy and thrombosis: reduced nitric oxide bioactivity and increased hemolysis (Kato et al., 2006).

In addition to the vasculopathy that underlies PH, a hypercoagulable state is also postulated to be present (Bazan and Fares, 2018). Patients are at an increased risk of developing thrombi. In pulmonary arterial hypertension, there is dysregulation of the coagulation cascade and fibrinolytic system. Postmortem studies reveal a high prevalence of vascular thrombotic lesions—the dysregulation of the fibrinolytic system characterized by increased plasma levels of fibrinogen and fibrinopeptide A- and D-dimers.

The epithelial dysfunction due to the underlying vasculopathy leads to increased plasma levels of von Willebrand factor and plasminogen activator inhibitor type 1, leading to a procoagulant state. The shear effect of the blood on the dysfunctional epithelial surface potentially generates a thrombogenic surface, resulting in increased thrombosis. Tissue factor is a glycoprotein that initiates a coagulation cascade and binds to factor VII to activate factor X. Studies have shown upregulation of tissue factor, and another study found increased tissue factor expressing endothelial microparticles in the circulation.

Our study has important strengths and limitations. The large sample size is a significant strength. In addition, a diverse representation of patients allows for population analysis. Certain limitations are to be considered. Since it is a retrospective study, particular inherent biases come with the study design. The database depends on the use of appropriate ICD-9 coding. So, inconsistencies in coding could have led to missing data. The sample represents about 20% of the admissions, and the sampled discharges within a state are variable. This could lead to underrepresentation and under-sampling. We included data from a single calendar year and could not assess the recent trend.

## Conclusion

Pulmonary hypertension is a crucial comorbidly for inpatients, which can potentially lead to increased hospital stay and worse outcomes. In our study, we found that black patients had more extended hospital stays, were younger and Hispanic patients were likely to die in the hospital. Individualized treatment strategies to identify the associated comorbidities can potentially lead to better outcomes for patients admitted with ischemic stroke. More animal models are needed to assess the intricate relationship between stroke and pulmonary hypertension. More clinical trials are needed to assess long-term mortality and morbidity and the effects of antithrombotics on the outcome. More studies is needed to understand the racial differences in the outcomes of patients with ischemic stroke and pulmonary hypertension.

## Data availability statement

The original contributions presented in the study are included in the article/supplementary material, further inquiries can be directed to the corresponding author.

## Author contributions

DJ: Conceptualization, Investigation, Writing – original draft, Writing – review & editing. SK: Formal analysis, Investigation, Methodology, Writing – original draft. GM: Writing – original draft. DR: Conceptualization, Validation, Investigation, Resources, Writing – original draft, Writing – review & editing.
